# Saprotrophic soil fungi to improve phosphorus solubilisation and release: In vitro abilities of several species

**DOI:** 10.1007/s13280-017-0972-0

**Published:** 2017-11-20

**Authors:** Andrea Ceci, Flavia Pinzari, Fabiana Russo, Oriana Maggi, Anna Maria Persiani

**Affiliations:** 1 Dipartimento di Biologia Ambientale, Sapienza Università di Roma, Piazzale Aldo Moro 5, 00185 Rome, Italy; 2 Consiglio per la ricerca in agricoltura e l’analisi dell’economia agraria. Centro di ricerca Agricoltura e Ambiente (CREA-AA), Via della Navicella 2-4, 00185 Rome, Italy

**Keywords:** Biofertilisers, Biomineralisation, Indices P solubilisation, Phosphorus, Soil saprotrophic fungi, Tricalcium phosphate

## Abstract

Modern agriculture is dependent on phosphate rock (PR), which is a nonrenewable resource. Improvement of phosphorus (P) availability for crops in agricultural soils represents a key strategy to slow down the depletion of PR. The aim of this study was to identify potential P biofertilisers among saprotrophic fungal species. We tested 30 fungal strains belonging to 28 taxa (4 Zygomycota and 24 Ascomycota) and with different life strategies. The study showed that many saprotrophic fungi have the ability to mobilise P from insoluble forms according to a variety of mechanisms. Our results expand the pool of P solubilising fungal species, also suggesting a new solubilisation index and shedding light on parameters that could be basic in the selection of efficient soil P-biofertilisers fungi. *Rhizopus stolonifer* var. *stolonifer*, *Aspergillus niger* and *Alternaria alternata* were found to be the best performing strains in terms of amounts of TCP solubilisation.

## Introduction

Global climate change and fast increase in the world population require a more efficient agriculture and food production. Modern agriculture and intensive crop production increase the demand for fertilisers (Rouached 2016). The world demand for phosphatic fertilisers is forecasted to grow annually by 2.2% between 2014 and 2018 (FAO 2015). However, modern agriculture is dependent from phosphate rock (PR), which is a non-renewable resource. In fact, current global reserves may be depleted in 50–100 years (Gunther 2005) and the fertilisers industry recognises that the quality of reserves is declining while the cost of extraction, processing and shipping is increasing (Cordell et al. 2009). One recent European Union’s report underlined the future phosphorus (P) limitation as one of the main reasons for the vulnerability of the European food system (Rouached 2016). Responses should include a more efficient production and use of P resources, as well as a greater awareness to avoid over-fertilisation with resulting environmental problems (e.g. eutrophication). A more integrated and effective approach to manage the P cycle is needed (Cordell et al. 2009). In this context, biofertilisation could have a positive role since it is based on the use of natural inputs including decaying remains of organic matter, animal manure, and the use of microorganisms such as fungi and bacteria (Carvajal-Muñoz and Carmona-Garcia 2012). A biofertiliser is a substance which contains living microorganisms which, when applied to soil, promote plants’ growth by increasing the supply or availability of primary nutrients.

The use of phosphate-solubilising microorganisms (bacteria and fungi) that break down inorganic soil phosphates to simpler forms thus enabling uptake by plants has been widely suggested (Saxena et al. 2014; Malusà et al. 2016). This approach has the potential not only to reduce the use of PR and its derivatives, but also improve the solubilisation of insoluble P minerals in a cheap, environmentally friendly and sustainable way, basically promoting plant growth by optimisation of stable soil P sources use. Fungi, in particular, resulted to be more efficient than bacteria in P solubilisation, on both solid agar and in liquid cultures (Kucey 1983; Halder et al. 1990; Saxena et al. 2014). Among the fungi, mycorrhizas are already exploited to improve P availability to plants as plant growth-promoting microorganisms (PGPM) (Owen et al. 2015). Arbuscular mycorrhizal (AM) fungi are vital components of nearly all terrestrial ecosystems, forming mutualistic symbioses with the roots of around 80% of vascular plants, thereby increasing phosphate uptake and growth. The mycorrhizas greatly increase the absorptive area of a plant, acting as extensions to the root system.

Mycorrhizas are vital in gathering P in uncultivated soils but in agricultural realities not all the crops can form mycorrhizas, and despite of mycorrhization in some soils, optimal P quantity for sustaining productivity may not be found (Oehl et al. 2004). Moreover, phosphorus-rich fertilisers suppress the mycorrhiza formation. Studies in which the soil P has been labelled with radioactive ^32^P indicated that both mycorrhizal and non-mycorrhizal plants utilised the similarly labelled P sources in soil (Bolan 1991). In many soils the main issue is the solubility of P and its slow release in the right forms to be absorbed by the plants’ roots.

Studies on the potential of soil saprotrophic fungi in P release from insoluble forms in soil are still scarce and only a few species have been studied, with a predominance of *Aspergillus* and *Penicillium* species. *Aspergillus niger* is a very efficient organic acid producing fungus and has been described in many studies as a strong P solubiliser (Pradhan and Sukla 2006; El-Azouni 2008; Bojinova et al. 2008; Pinzari et al. 2012). Other fungal genera successfully tested by some authors for their phosphate solubilisation ability are *Trichoderma*, *Mucor*, *Candida*, *Discosia*, *Eupenicillium, Gliocladium* and some species of yeasts (Rosling et al. 2007; Jain et al. 2012; Saxena et al. 2014). The mechanisms of PR solubilisation by saprotrophic soil fungi can be very diversified between species, for example entailing the production of different combinations of organic acids such as oxalic and citric, in order to attack the PR structure and increase its solubility. The potential of some species to be used as P-solubilisation helpers in poor or highly exploited soils is still untapped. On the other side, fungi can also mediate the formation of new secondary minerals of P, reducing the P loss from soil because of surface run off (Burford et al. 2003; Fomina et al. 2005).

The use of P solubilising fungi in agriculture could be addressed also to the treatment of agro-industrial wastes and the production of organo-mineral fertilisers, where mineral P is partially immobilised into biomass and could be slowly released in soil. In many countries exhausted fungal biomasses are used as fertilisers. The Italian law, for example, allows the use of exhausted fungal material as N-P fertiliser if it contains at least 1% P_2_O_5_ (Italian legislative decree 29th April 2010, n. 75 and its modifications). Therefore, the selection of fungi capable of solubilising or storing P could have an application in the vast field of fertiliser production and improvement.

The aim of this study was to select strains that, when applied to agricultural soil, could promote the growth of plants by improving the supply or availability of P. We tested 30 fungal strains belonging to 28 taxa (4 Zygomycota and 24 Ascomycota) with different life strategies on Pikovskaya’s medium containing 5% tricalcium phosphate (TCP). The evaluation of secondary minerals precipitation by fungi and the strategies of P uptake by fungal biomasses were investigated at a microscale level, using Scanning electron microscopy (SEM) coupled with energy-dispersive X-ray spectroscopy (EDS). SEM has been used extensively for the visualisation of fungi in association with minerals (Fomina et al. 2005; Burford et al. 2006; Rosling et al. 2007) because it can provide spatial information at the micrometer to sub-micrometer scales and when used with EDS it can reveal high-resolution elemental compositional measurements. The presence of secondary minerals formation in the floating fungal mycelium was documented and their elemental composition evaluated by EDS.

## Materials and methods

### Solubilisation test in PVK agar medium

Thirty strains of saprotrophic fungi were tested for their P solubilisation and uptake ability. They are currently preserved at the culture collection of the Fungal Biodiversity Lab (FBL) (Sapienza, University of Rome) (Table 1). The selected strains were representative taxa of different phyla. The strains were isolated in previous studies mostly from soil and from different geographical areas, such as Mediterranean maquis soils (e.g. *Minimedusa polyspora*) and tropical forest soils (e.g. *Heterocephalum taiense*) (Persiani and Maggi 1986; Pinzari et al. 2014). Many of the isolates belong to either genera or species known for their ability in mineral P solubilisation. This is the case for *Aspergillus*, *Rhizopus*, *Alternaria*, and *Penicillium*. In particular, *A. niger* was tested as a positive control, to be compared with the other species. The selected strains possess different physiological and metabolic features. Some of them can play several ecological functions in ecosystems. For instance, *Trichoderma harzianum* is reported to have biocontrol properties, which could be a further element of interest (Vassilev et al. 2006).

**Table 1:** List of the 30 fungal strains tested in solid culture on Pikovskaya’s medium containing 5% insoluble tricalcium phosphate as only P source. From left to right in columns: growth diameter (mm) of the fungal colony, halo zone (mm) measured under the colony after the removal of the cellophane membrane, fungal biomass expressed as dry weight (g), average pH of the Pikovskaya’s medium after fungal growth. The values are reported as averages ± Standard deviation out of three biological replicas. Within each replicas (i.e. plate/colony) at least 10 measures were effected to obtain a single average value in the case of diametrical data because of the variability of the shape that fungal colonies can assume according to the species and growth peculiarities

Fungal strains	Growth diameter (mm)	Halo zone (mm)	Dry weight (g)	pH
*Alternaria alternata* (Fr.) Keissl. FBL 508	64.0 ± 3.6	26.7 ± 10.4	0.094 ± 0.004	4.39 ± 0.17
*Alternaria* sp. FBL 507	55.0 ± 5.0	8.3 ± 1.5	0.027 ± 0.005	4.50 ± 0.17
Anamorphic fungus FBL 165	35.0 ± 0.0	8.7 ± 15.0	0.025 ± 0.004	3.72 ± 0.23
*Arthrinium arundinis* (Corda) Dyko & B. Sutton FBL 266	58.0 ± 7.6	3 ± 5.2	0.011 ± 0.001	4.69 ± 0.55
*Aspergillus niger* Tiegh. FBL 453	72.0 ± 7.2	73.3 ± 5.8	0.136 ± 0.009	1.53 ± 0.07
*Aspergillus* sp. FBL 253	16.0 ± 1.7	0 ± 0	0.036 ± 0.059	5.36 ± 0.32
*Cadophora fastigiata* Lagerb. & Melin FBL 557	21.0 ± 1.7	0 ± 0	0.004 ± 0.002	5.93 ± 0.45
*Cladosporium* sp. FBL 513	11.0 ± 1.7	0 ± 0	0.004 ± 0.002	6.93 ± 0.23
*Cladosporium tenellum* K. Schub, Zalar, Crous & U. Braun FBL 505	27.3 ± 2.5	4.0 ± 3.6	0.044 ± 0.010	4.33 ± 0.16
*Engyodontium album* (Limber) de Hoog FBL 458	24.3 ± 0.6	6.0 ± 0.0	0.025 ± 0.005	4.43 ± 0.41
*Epicoccum nigrum* Link FBL 181	45.0 ± 5.0	10.7 ± 0.6	0.013 ± 0.000	4.62 ± 0.26
*Heterocephalum taiense* Persiani & Maggi FBL 249	21.3 ± 5.5	16.7 ± 0.6	0.019 ± 0.002	4.26 ± 0.49
*Microdiplodia* sp. FBL 512	25.0 ± 5.0	4.0 ± 3.6	0.008 ± 0.001	6.18 ± 0.37
*Minimedusa polyspora* 7 (Hotson) Weresub & P. M. LeClair FBL 503	29.0 ± 1.7	5.7 ± 5.1	0.015 ± 0.004	4.54 ± 0.29
*Minimedusa polyspora* 5b (Hotson) Weresub & P. M. LeClair FBL 514	29.0 ± 3.6	4.7 ± 4.0	0.011 ± 0.001	5.06 ± 0.17
*Minimedusa polyspora* 5a (Hotson) Weresub & P. M. LeClair FBL 502	24.0 ± 2.0	6.7 ± 2.5	0.010 ± 0.000	5.07 ± 1.02
*Mortierella globalpina* W. Gams & Veenb.-Rijks FBL 74	30.0 ± 3.6	11.7 ± 2.9	0.028 ± 0.005	4.30 ± 0.18
*Myrothecium cinctum* (= *Striaticonidium cinctum* (Corda) L. Lombard & Crous) FBL 383	41.7 ± 2.9	0 ± 0	0.019 ± 0.002	4.78 ± 0.29
*Myrothecium verrucaria* (= *Albifimbria verrucaria (*Alb. & Schwein.) L. Lombard & Crous) FBL 385	24.0 ± 1.0	5.0 ± 1.7	0.010 ± 0.001	5.16 ± 0.29
*Oidiodendron* sp. FBL 319	15.0 ± 0.0	3.0 ± 5.2	0.008 ± 0.001	5.23 ± 1.46
*Paecilomyces lilacinus* (= *Purpureocillium lilacinum* (Thom) Luangsa-ard, Houbraken, Hywel-Jones & Samson) FBL 320	26.0 ± 6.9	12.3 ± 5.5	0.018 ± 0.004	4.26 ± 0.61
*Paecilomyces marquandii* (= *Metarhizium marquandii* (Massee) Kepler, S. A. Rehner & Humber) FBL 321	32.7 ± 2.3	11.0 ± 1.0	0.052 ± 0.029	4.89 ± 0.72
*Paecilomyces variotii* Bainier FBL 213	29.0 ± 1.0	0 ± 0	0.008 ± 0.002	5.82 ± 0.19
*Penicillium griseofulvum* Dierckx FBL 500	52.0 ± 7.2	15.7 ± 4.01	0.080 ± 0.011	4.42 ± 0.72
*Phycomyces nitens* (C. Agardh) Kunze FBL 504	59.0 ± 7.9	4.7 ± 0.6	0.012 ± 0.002	4.38 ± 1.18
*Rhizopus stolonifer* var. *stolonifer* (Ehrenb.) Vuill. FBL 256	86.0 ± 0.0	86.0 ± 0.0	0.051 ± 0.013	3.61 ± 0.13
*Stilbella* sp. FBL 501	21.3 ± 1.5	3.0 ± 5.2	0.006 ± 0.001	5.71 ± 0.10
*Trichoderma harzianum* Rifai FBL 365	86.0 ± 0.0	1.7 ± 2.9	0.028 ± 0.006	4.77 ± 0.23
*Umbelopsis nana* (Linnem.) Arx FBL 77	23.3 ± 2.9	0 ± 0	0.006 ± 0.002	6.53 ± 0.13
*Volutella* sp. FBL 369	20.3 ± 0.6	12 ± 1	0.011 ± 0.001	5.07 ± 0.43

Among the tested strains, a few of them, like *A. niger*, *Cladosporium* sp. and *A. alternata* are common soil saprotrophic species that in some conditions can behave as pathogens for humans and/or plants. These were chosen since their r-selected life-style characterizes them as fast-growing species with high potential for biomass production. Exhausted and sterilised fungal biomasses containing organic forms of P could be, in fact, of interest in agriculture, where slow release organo-mineral fertilisers could find a market.

The strains were reactivated and routinely maintained on malt extract agar (MEA, Difco, Sparks, MD, USA). Pikovskaya (PVK) agar with 5% of tricalcium phosphate [Ca_3_(PO_4_)_2_] (TCP) (Pikovskaya 1948; Nautiyal 1999) was employed to screen phosphate solubilising microorganisms by a plate assay method. Therefore, the only P source was represented by the insoluble TCP. The pH of the media, not adjusted, was 6.5 ± 0.2. For the experiments sterile cellophane membranes (Focus Packaging and Design Ltd, Louth, UK) were used according to Sayer and Gadd (1997) and to Ceci et al. (2015). The fungal strains were grown for at least 7 days on MEA at 25 °C in the dark prior to experimental subculture. Inoculation was carried out by using 5 mm diameter cores of mycelium taken from actively growing margins of stock colonies by using a sterile cork borer. Fungal growth was evaluated by diametric extension and by the biomass yield (Vodnik et al. 1998). After 7 days, fungal colonies were removed from the agar by peeling the biomass from the cellophane membranes using a sterile razor blade. Mycelia were oven-dried at 100 °C until reaching constant weight for 2 days. After the biomass removal, the surface pH of the agar was measured at specific intervals across the diameter of the Petri dish using the pH portable meter for food and dairy HI 99161, fitted with a conical tip FC 202D pH electrode (Hanna Instruments, Woonsocket, RI, USA). The ability of different strains to solubilise phosphate was determined after 7 days of incubation at 25 °C in the dark by employing two different indices. The solubilisation index (SI) was calculated according to Gudiño Gomezjurado et al. (2015). Moreover, a further index has been proposed and used in this research, taking account the measures of the diameter of the fungi, the diameter of the halo zone around the growing colonies and the average medium pH. The solubilisation—pH index (SpHI) was calculated according to the following formula:

$$ {\text{SpHI}} = {\text{SI}} \times \left( {\overline{{{\text{pH}}_{\text{C}} }} - \overline{{{\text{pH}}_{\text{T}} }} } \right) $$

where SI is the solubilisation index above-described, and pH_C_ and pH_T_ are the average values of pH at control (PVK without fungus) and test, respectively.

Among the species previously tested in PVK solid medium a subset was chosen to be studied in PVK liquid medium for further investigations in different cultural conditions (Table 3).

The solubilisation index (SI) (Gudiño Gomezjurado et al. 2015), used to evaluate the solubilisation potential of tested fungal strains, takes account of both the halo zone formation and the diametric extension. This index is based on parameters of fungal morphology and growth. While SpHI index, proposed in this research, integrates the previous parameters with the lowering of medium pH which is related to fungal metabolic activity. Hence, the SpHI is a more comprehensive index, which may help to better select P-solubilisers.

### Solubilisation test in PVK broth medium

The experiments on PVK broth medium were carried out to evaluate the responses in these cultural conditions of the studied species and by performing observations on fungal mycelium and elemental analyses on biomass samples using SEM/EDS. In this way, it was possible to better investigate the biotransformation of TCP by fungi without the interferences of agar.

Solubilisation activity was carried out in 35 ml PVK broth medium in 150 ml sterile containers at 25 °C in the dark. Inoculation was carried out as described above. The pH of the medium was not adjusted and was 6.4 ± 0.0. Fungi were grown floating on the surface of the liquid PVK by the means of a perforated plastic support, in order to prevent the contact with TCP precipitated at the bottom of the containers. The fungal mycelia were harvested from liquid PVK by filtration using filter paper (Whatman—Rapida A Perfecte, Milan, Italy) and washing twice with distilled sterile water. Mycelia were oven-dried to estimate the dry weights. Fresh biomass subsamples and TCP were collected to be analysed by SEM/EDS before drying. The pH of liquid PVK was analysed for all tested fungi.

### Scanning electron microscopy (SEM)-energy-dispersive X-ray spectroscopy (EDS)

The fresh biomass of nine fungal species was examined uncoated, after the static liquid experiments using a variable pressure SEM (EVO50, Carl-Zeiss Electron Microscopy Group) equipped with a detector for backscattered electrons (BSE). Chemical analysis was performed by means of EDS (INCA 250, Oxford). The SEM was fitted with a tungsten filament and operated at 20 keV, with an average working distance of 12.5 mm, and with a chamber pressure between 30 and 150 Pa, chosen according to the need for maintenance of fungal turgidity. The EDS analyses were calibrated using standards (CaCO_3_, SiO_2_, Albite, MgO, Al_2_O_3_, GaP, FeS_2_, Wollastonite, MAD-10 Feldspar, Ti and Fe), and the conventional ZAF correction (for atomic number Z, absorption and fluorescence) was applied, integrated into the Oxford INCA 250 microanalysis package used (Burford et al. 2003).

In the SEM–EDS analysis, the fungal biomass which developed on the surface of the static cultures was sampled with plastic tweezers and deposited on aluminium stubs (12.5 mm, Agar Scientific) fitted by a sulphur free carbon adhesive (Agar Scientific). EDS spectra were acquired pointing at fungal mycelium and at the biogenic minerals eventually formed within the biomass. Two separate datasets were organised in order to obtain information on the elemental composition of the mycelium and the minerals that it concentrated and eventually precipitated.

### Statistical analysis

One-way analysis of the variance (ANOVA) was firstly applied on diametric colony extension, biomass yield, halo zones diametric extension and pH, and the significance of the differences was tested at 95% confidence. ANOVA was followed by stepwise multiple comparisons using the Newman-Keuls (SNK) method. Moreover, principal component analysis (PCA) was performed on these four variables. All the experiments were run in triplicate. ANOVA was also applied on the EDS measurements to evaluate the relationships between variables (e.g. elements, fungal strain, crystals) and the significance of the differences between samples. The variables were the 9 chemical elements analysed (C, O, Na, Ca, Mg, K, Cl, S, P). In this case, the ANOVA model used was “unbalanced” because the number of observations within each category was not the same. ANOVA was followed by a post hoc analysis using Tukey, and Bonferroni correction procedure in determining the critical value for significance. Statistical analyses of the SEM–EDS chemical data were used to investigate if it was possible to discriminate between fungal routes of TCP dissolution and P uptake mechanisms. PCA was then applied to the SEM–EDS results to study and visualise the correlations among all the variables (Massart et al. 1998). The statistical package XLStat (Addinsoft 2007-Pro, Paris, France) was used for all the statistical processing.

## Results

### Solubilisation test in PVK agar medium

All tested fungi were able to grow on PVK solid medium. *R. stolonifer* var. *stolonifer* and *T. harzanium* were the best performing fungi, showing the highest average diametric extension values and the highest overall growth rates of colonies (Table 1). ANOVA revealed that the growth extension for these fungi was significantly higher than all the other strains and control (*p* < 0.001). With regard to the diametric extension of the solubilisation halo zones, *R. stolonifer* var. *stolonifer*, *A. niger* and *A. alternata* resulted the best performing strains (Table 1). The halo zones formed by these strains were significantly larger than for all the other species. *A. niger*, *R. stolonifer* var. *stolonifer* and *H. taiense* showed the highest Solubilisation Index (0.7 > SI ≥ 1) (Table 2). *A. niger, A. alternata, P. griseofulvum, P. marquandii, R. stolonifer* var. *stolonifer* showed the highest biomass yields which were significantly higher than all the tested strains (*p* < 0.001) (Table 1). All fungi were able to lower the initial pH of PVK medium. *A. niger* and *R. stolonifer* var. *stolonifer* showed the lowest pH values, which were significantly different from those of the other tested strains (*p* < 0.01) (Table 1). *A. niger*, *R. stolonifer* var. *stolonifer, H. taiense* and *P. lilacinus* showed the highest SpHI values (SpHI > 1) (Table 2). This index was used to select the nine fungal strains among all to carry out experiments in liquid PVK.

**Table 2:** Solubility indexes calculated on the 30 fungal strains according to the variables reported in Table 1. The Solubilisation Index (SI) was calculated according to the ratio of the total diameter (colony + halo zone) and the colony diameter (El-Azouni 2008). The Solubility pH Index (SpHI) is here proposed for the first time and respect to the other index also considers the average agar pH at the end of incubation

Fungal strains	Acronyms	S.I.	SpHI
*Alternaria alternata* FBL 508	Alt1	0.42	0.87
*Alternaria* sp. FBL 507	Alt2	0.15	0.30
Anamorphic fungus FBL 165	Anm	0.25	0.68
*Arthrinium arundinis* FBL 266	Arth	0.05	0.09
*Aspergillus niger* FBL 453	Asp1	1.02	4.97
*Aspergillus* sp. FBL 253	Asp2	0.00	0.00
*Cadophora fastigiata* FBL 557	Cado	0.00	0.00
*Cladosporium* sp. FBL 513	Clad1	0.00	0.00
*Cladosporium tenellum* FBL 505	Clad2	0.15	0.31
*Engyodontium album* FBL 458	Engy	0.25	0.51
*Epicoccum nigrum* FBL 181	Epic	0.24	0.44
*Heterocephalum taiense* FBL 249	Hete	0.78	1.73
*Microdiplodia* sp. FBL 512	Micr	0.16	0.05
*Minimedusa polyspora 7* FBL 503	Min1	0.20	0.38
*Minimedusa polyspora* 5b FBL 514	Min2	0.19	0.26
*Minimedusa polyspora* 5a FBL 502	Min3	0.28	0.39
*Mortierella globalpina* FBL 74	Mort	0.39	0.85
*Myrothecium cinctum* FBL 383	Myr1	0.00	0.00
*Myrothecium verrucaria* FBL 385	Myr2	0.21	0.27
*Oidiodendron* sp. FBL 319	Oidi	0.20	0.25
*Paecilomyces lilacinus* FBL 320	Pae1	0.47	1.05
*Paecilomyces marquandii* FBL 321	Pae2	0.34	0.54
*Paecilomyces variotii* FBL 213	Pae3	0.00	0.00
*Penicillium griseofulvum* FBL 500	Peni	0.30	0.62
*Phycomyces nitens* FBL 504	Phy	0.08	0.17
*Rhizopus stolonifer* var. *stolonifer* FBL 256	Rhiz	1.00	2.87
*Stilbella* sp. FBL 501	Stil	0.13	0.10
*Trichoderma harzianum* FBL 365	Tric	0.02	0.03
*Umbelopsis nana* FBL 77	Umb	0.00	0.00
*Volutella* sp. FBL 369	Volu	0.59	0.83

**Fig. 1:**
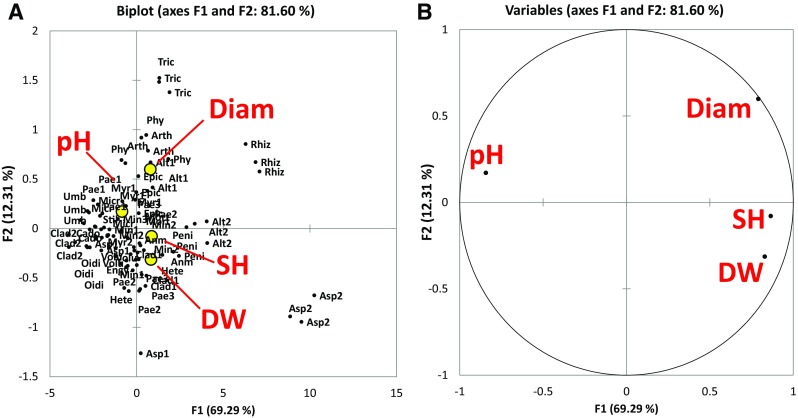
Principal component analysis (Pearson). **A** Plot of the two principal components obtained from the data of diametric extension (DIAM), pH, diametric extension of solubilisation halo zones (SH) and biomass yield (DW). **B** Correlation plot between loadings and variables. Bartlett sphericity test for the first two components: the computed *p* value is > 0.001, lower than the significance level alpha = 0.05, thus we accepted the hypothesis Ha (at least one of the correlations between the variables is significantly different from 0). The correlation between the variables is reported and discussed in the text

The values of diametric extension (DIAM), pH, diametric extension of solubilisation halo zones (SH) and biomass yield (DW) varied consistently among the 30 strains. They showed some statistically significant correlations (Fig. 1) and some expected autocorrelations that, however, can be an expression of the underlying mechanisms in TCP solubilisation by fungi with different genotypes and phenotypes. In particular, positive correlation between diametrical extension and biomass yield (*R* = 0.522, *p* < 0.001), positive correlation between diametrical extension and solubilisation halo zone (*R* = 0.632, *p* < 0.001) and negative correlations between all the growth parameters (DW, SH and Diam) and the pH were observed. The strongest negative correlation was referred to pH and the solubilisation halo (*R* = − 0.648, *p* < 0.001). In the PCA plot (Fig. 1) the first component (F1) accounted for the 69.29% of data variability while the second component accounted for the 12.31% of the variability.

In the ordination space, the fungal species observations were mainly distributed in the centre of the plot, except a few outliers, such as *Aspergillus* sp. FBL 253, *R. stolonifer* var. *stolonifer*, *Alternaria* sp., *C. tenellum*, *P. nitens*, *A. arundinis* and *T. harzianum* (Fig. 1).

### Solubilisation test in PVK broth medium

*A. niger*, *H. taiense* and *M. globalpina* showed the highest biomass yields, which were significantly higher than all tested strains (*p* < 0.001) (Table 3). All fungi were able to lower the initial pH of PVK broth medium. *A. niger*, *P. griseofulvum* and *R. stolonifer* var. *stolonifer* showed the lowest pH values, which were significantly different from those of the other tested strains (*p* < 0.001) (Table 3).

**Table 3:** The nine fungal strains selected among the 30 tested in solid culture to be essayed in static liquid experiments on Pikovskaya’s medium containing 5% insoluble tricalcium phosphate as only P source. The table reports the fungal biomass dry weights and the pH of the liquid medium at the end of the incubation. The values are reported as averages ± Standard deviations from ten biological replicas

	Fungal strains	Dry weight (g)	pH
1	*Mortierella globalpina*	0.285 ± 0.013	5.09 ± 0.08
2	*Minimedusa polyspora*	0.019 ± 0.001	5.75 ± 0.13
3	*Rhizopus stolonifer* var. *stolonifer*	0.195 ± 0.011	3.96 ± 0.11
4	*Paecilomyces marquandii*	0.129 ± 0.010	5.60 ± 0.27
5	*Phycomyces nitens*	0.013 ± 0.005	6.36 ± 0.12
6	*Heterocephalum taiense*	0.322 ± 0.014	5.05 ± 0.16
7	*Paecilomyces lilacinus*	0.182 ± 0.025	5.18 ± 0.29
8	*Penicillium griseofulvum*	0.193 ± 0.037	3.39 ± 0.11
9	*Aspergillus niger*	0.311 ± 0.019	2.04 ± 0.18

### Scanning electron microscopy (SEM)

The fungus that accumulated more P in the biomass was *R. stolonifer* var. *stolonifer* (Table 4), followed by *P. griseofulvum* and *A. niger*. *R. stolonifer* var. *stolonifer* precipitated also secondary containing P minerals in its biomass.

**Table 4:** Phosphorus (P) and Calcium (Ca) average concentration (wt%) in the fungal biomass floating on the surface of the static cultures (Fungal Biomass), and P and Ca content in the biogenic minerals found embedded in the same fungal biomasses (Biogenic minerals) in samples 1–9, corresponding to as many fungal species. The data were obtained with energy-dispersive X-ray spectroscopy (EDS), from different areas of the fungal biomass as visualised with a variable pressure scanning electron microscope equipped with a backscattered electron detector, which permitted to distinguish between embedded minerals and fungal structures. Sample 7 did not show any biogenic mineral in the suspended mycelium. The numbering of the 9 species is the same used in the PCA plots of Fig. 3

		P biomass	P biogenic minerals	Ca biomass	Ca biogenic minerals
1	*M. globalpina*	1.1 ± 0.2^e^	1.6 ± 0.1^b,c^	0.3 ± 0.2^d^	0.5 ± 0.2^c^
2	*M. polyspora*	0.9 ± 0.3^e^	0.2 ± 0.1^c^	1.3 ± 2.6^d^	15.6 ± 6.5^a^
3	*R. stolonifer* var. *stolonifer*	4.7 ± 2.3^a^	6.4 ± 1.2^a^	11 ± 4.8^a^	12.7 ± 2.5^a,b^
4	*P. marquandii*	1 ± 0.5^e^	2.4 ± 1^b^	1 ± 0.9^d^	3.8 ± 2^c^
5	*P. nitens*	1.9 ± 0.6^c,d^	3.4 ± 2.7^a,b^	4.2 ± 2.2^c^	10.3 ± 7.1^b^
6	*H. taiense*	1 ± 0.4^e^	4 ± 1.7^a,b^	0.8 ± 0.6^d^	7.1 ± 4.3^b,c^
7	*P. lilacinus*	1.4 ± 0.3^d,e^	nd	0 ± 0^d^	nd
8	*P. griseofulvum*	2.4 ± 1.4^b,c^	2.3 ± 1.5^b^	6.9 ± 7.3^b^	19.4 ± 9.8^a^
9	*A. niger*	2.7 ± 1.3^b^	2.1 ± 1.1^b,c^	0.9 ± 1.3^d^	6.3 ± 2.4^b,c^

Different letters (from “a” to “e”) indicate significant differences (ANOVA post hoc analysis, *p* < 0.05) between the species for the investigated parameters. *nd* indicates no values could be determined

**Fig. 2:**
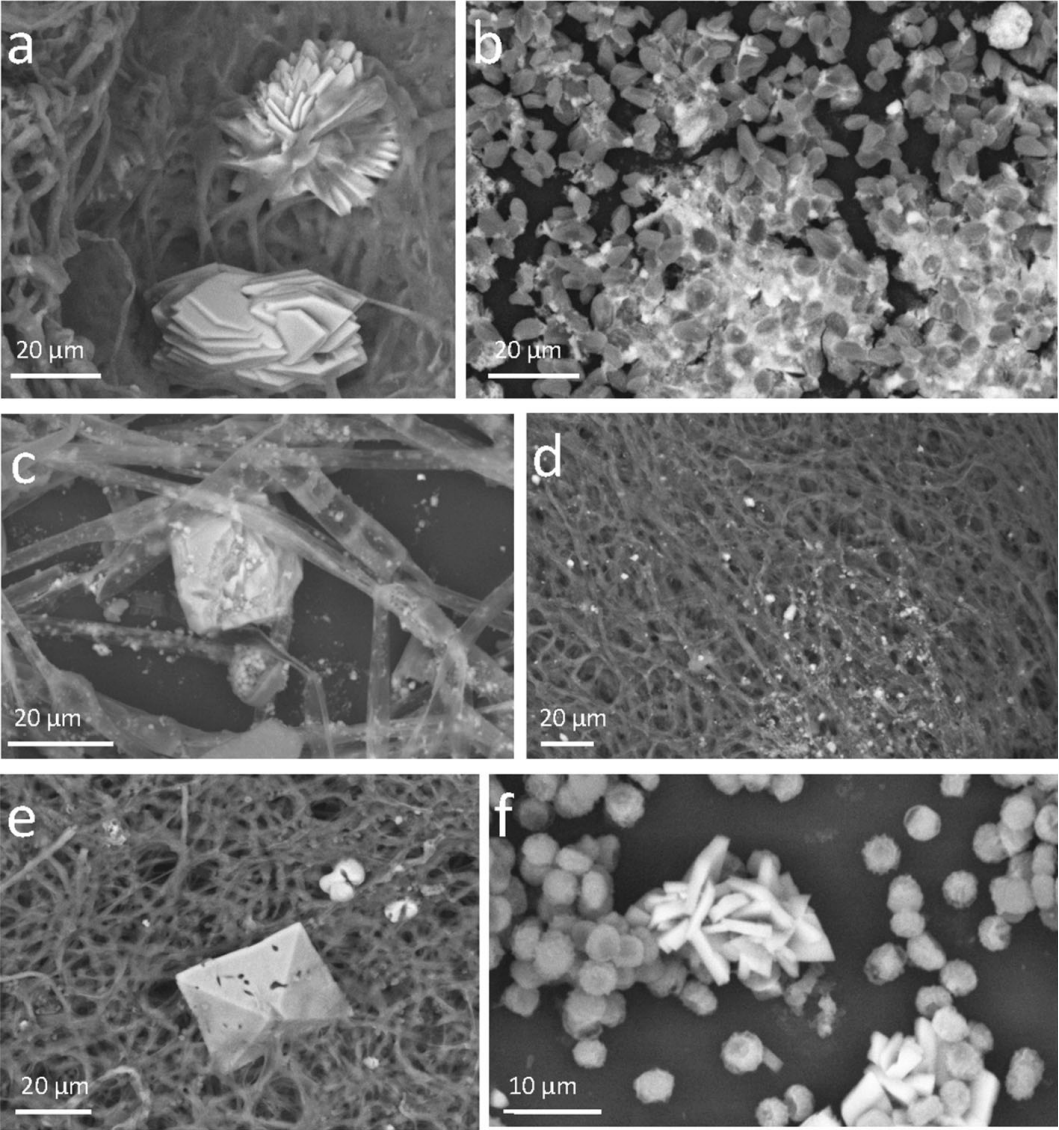
VP-SEM-BSD images of fungal biomass with embedded secondary biogenic crystals or mineral precipitates. The images were obtained observing not fixed or metallised fresh fungal material at a pressure of about 60 Pa. The objects made of elements with a higher atomic number appear lighter than organic ones. Backscattered electrons, in fact, return chemical images of what scanned. **a** *Minimedusa polyspora*, **b** *Rhizopus stolonifer* var. *stolonifer*, **c** *Phycomyces nitens*, **d** *Heterocephalum taiense*, **e** *Penicillium griseofulvum*, **f** *Aspergillus niger*

Other fungi that promoted the accumulation of P containing minerals in the floating fungal biomass were *P. nitens* and *H. taiense*. *R. stolonifer* var. *stolonifer* was able also to store calcium (Ca) both in its hyphae and in deposited biogenic minerals. Other species that were effective in absorbing Ca in the mycelium were *P. nitens* and *P. griseofulvum*. The precipitation of biogenic minerals based on Ca was massive in many species (Table 4). Biogenic minerals containing a high Ca concentration were produced in the fungal biomass by almost all the species and in particular by *P. griseofulvum*, *M. polyspora*, *R. stolonifer* var. *stolonifer*, and *P. nitens* (Fig. 2, Table 4).

Distinctive calcium oxalates crystals (Fig. 2), both in the bipyramidal form of CaC_2_O_4_(2 + X)·H_2_O and in the monohydrate form CaC_2_O_4_·H_2_O were observed in some of the fungal biomasses grown at the presence of TCP. Oxalate crystals exhibited a variety of crystalline forms (bipyramidal, plate-like, druses, rhombohedral, etc.).

**Fig. 3:**
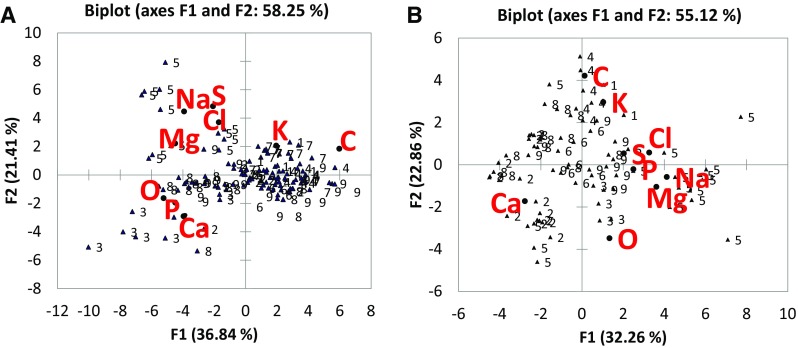
Principal component analysis (Pearson). **A** Plot of the two principal components obtained from the elemental composition dataset (EDS measurements) of fungal biomasses; **B** Plot of the two principal components obtained from the elemental composition dataset (EDS measurements) of crystals and mineral precipitates embedded in fungal biomasses

The PCA plots (Fig. 3) showed that in fungal mycelium (Plot A) and in the biogenic crystals and mineral precipitates embedded in the fungal biomasses, the correlation between microelements was different. In particular, the Plot A (elemental composition of fungal biomasses) showed a correlation between the uptake of P and Ca, with the X axis representing the concentration of elements (decreasing from left to right). While in the Plot B (elemental composition of crystals and mineral precipitates embedded in fungal biomasses) P and Ca were inversely correlated. All the elements in the plot, except Ca and P, came from the liquid media (PVK), while Ca and P resulted from the solubilisation of the added TCP.

In the Plot A, the species 3, 8 and 5 (*R. stolonifer* var. *stolonifer*, *P. nitens* and *P. griseofulvum*) were positively correlated to Ca and P. In Plot B the species correlated with Ca were those exhibiting calcium oxalate crystals (*M. polyspora*, *A. niger*, *P. griseofulvum*).

## Discussion

The results of our study showed TCP solubilisation by 30 fungal strains with different metabolisms and life-styles. The correlation between the extension of solubilisation halo and the pH values in the solid medium experiment suggested that one of the main mechanisms of TCP solubilisation may be the production and release of organic acids by fungi. This is corroborated by other studies that showed how fungal solubilisation of P compounds in liquid cultures is positively correlated to the amount of released acidic compounds (Gupta et al. 1994; Rosling et al. 2007). Nevertheless, some other mechanisms, such as enzymes production could have played a substantial role in the mechanisms put at place by the tested fungal strains to uptake P. The main mechanisms that fungi use to acidify their microenvironment can vary due to both the strains and the kind of substrate. These mechanisms could represent the main discriminant in the choice of the best strains to be used as a solubilisation aid in agricultural soils. In fact, fungi may induce TCP weathering also through cation biosorption to exchange sites in the cell wall (Marschner et al. 1998) and modulate uptake and availability of P by storing it in new mineral forms that precipitate in the mycelium (Rosling et al. 2007). The slow release of soluble P could represent a remedy for those agricultural soils that genetically promote P immobilisation, and render the use of phosphate fertilisers useless. According to Burford et al. (2003) fungi solubilise minerals, including phosphate rocks, with active secretion of organic acids, proton efflux out the plasma membrane, formation of carbonic acid in the media as a result of respiratory CO_2_ production. Fungi that produce a clear zone under their growing colony in a solid medium that contains insoluble calcium phosphate are phosphorus-solubilising organisms (Whitelaw 2000). According to this definition 6 strains out of the 30 tested did not result as P solubilisers. These six strains, however, did grow in the solid medium, despite the absence of available P. These species could have recycled and used the little amounts of P available from the same inoculum, and this could have sustained the growth of the mycelium for a short time. On the other side, it is not possible to exclude that these strains could have used some of the insoluble P present in TCP with unidentified mechanisms which granted them a P supply, although without visible effects in terms of minerals leaching. *M. cinctum,* for instance, produced a mycelium with an average diameter of 41.7 ± 2.9 mm, and a dry weight (0.019 ± 0.002 g) far higher than other species that conversely showed important solubilisation halos. Moreover, although *M. cinctum* showed some degree of acidification ability (pH 4.78 ± 0.29) it did not produce a solubilisation halo, highlighting that the link between halo formation on TCP and solubilisation ability a non-univocal one. It is possible that the kind of acid produced defines most the dissolution pattern of TCP in the media, despite the real need for P of the growing fungus. According to Rosling et al. (2007) in Mucorales exudates predominates the oxalic acid, which can induce both TCP and fluorapatite dissolution at high rates. Besides, some Ascomycetes induce P-minerals dissolution by lowering the pH of the media without producing significant amounts of low molecular weight organic acids (Rosling et al. 2007). The hypothesis formulated by Rosling et al. (2007), and somehow confirmed by our results, is that while low molecular weight organic acids are strong dissolution agents, they do not appear to be produced by all fungi in response to P-limiting growth conditions, because other equally efficient but not entirely revealed and explained mechanisms allow fungi to obtain P from limiting environments. It follows that the in vitro-screening of fungi performed by measuring only the solubilisation halo and the pH is inefficient in strains selection.

In our experiment, the more diffusible compounds, capable of lowering the pH of the medium, were produced by the fungi, and more TCP was solubilised under the mycelium. However, although there was this correlation, the value of R suggested that not for all species the mechanism was so straightforward, and that there was a gradient that placed some species in a different position. This could be seen in the PCA plot of Fig. 1 where the first component (F1) could be interpreted as the growth of the fungi, while the second component, was mostly associated to pH, indicating that these two fungal characteristics (fungal growth and lowering of pH) occurred independently.

The SpHI index, proposed in this research, integrated the halo zone formation and the diametric extension used in the solubilisation index (SI) (Gudiño Gomezjurado et al. 2015) with the lowering of medium pH, which is related to fungal metabolic activity, resulting in a more comprehensive and effective index for P-solubilisers selection.

Out of the nine species we tested more deeply, actually only four resulted phosphate solubilisers. The species *A. niger*, *P. lilacinus*, *P. marquandii* and *P. griseofulvum* showed a negative correlation between medium pH and solubilisation of P, according to other authors (Cerezine et al. 1988; Reddy et al. 2002; Wakelin et al. 2004; Maurya and Kumar. 2006; Hernández-Leal et al. 2011; Qiu and Lian 2012; Posada et al. 2013). For the genus *Mortierella,* P-solubilisation has been already described (Osorio and Habte 2001) but for the species *M. globalpina* a remarkable efficiency of insoluble phosphate solubilisation, coinciding with decrease in medium pH, has been showed for the first time.

The current study also showed that each of the nine fungal species used in the liquid media test, performed differently in terms of P uptake in the biomass and ability to precipitate secondary minerals in the mycelium. *R. stolonifer* var. *stolonifer*, *P. marquandii*, *P. nitens* precipitated P minerals in association with fungal mycelia. Biologically induced precipitation of hydroxyapatite has been demonstrated by Rosling et al. (2007) in Mucorales.

The multivariate output of EDS data (Fig. 3, plots A and B) showed that in fungal biomasses there was a correlation between the uptake of P and Ca while in mineral precipitates P and Ca were inversely correlated and Ca was released or taken up independently of P.

The presence of calcium oxalate crystals associated with mycelia was observed in some of the tested fungi. Distinct crystals were, in fact, documented mainly in *M*. *polyspora*, *A. niger*, and *P. griseofulvum*. The production of oxalates by fungi during apatite or TCP solubilisation was reported to be a massive mechanisms of passive weathering, namely a release of acidic compounds in the media regardless of the fungal needs of elements for growth (Rosling et al. 2007; Pinzari et al. 2013). Interestingly in some fungi, Ca and P supplementation significantly affected the amount of calcium oxalate formed (Shinners and Tewari 1997; Tuason and Arocena 2009). In the fungus *Piloderma fallax* (Lib.) Stalpers more calcium oxalate was formed at high P levels, with a strong positive linear relationship between Ca level and calcium oxalate but only under conditions of P limitation (Tuason and Arocena 2009). The in vitro testing of fungal strains clearly does not include some important variables that come into play under natural conditions. Some species of fungi, highly performing in vitro, may have a different behaviour in natural situations. Their potential to colonise mineral surfaces and their geoactive properties need to be verified in conditions closer to real situations.

## Conclusions

In this study, we described an optimisation of the steps and methods for evaluating the tricalcium phosphate solubilising strength of fungi. In particular, the chemical data, obtained analysing fungal mycelia with SEM–EDS technique, were used to discriminate between fungal routes of TCP dissolution and P uptake mechanisms.

The solubilisation halo and the lowering of the pH, taken individually, represented weak indicators of the real fungal potential to make P bioavailable to plants in soil. The results of our study showed that saprotrophic fungi can modulate uptake and availability of P by different mechanisms. In particular, they can store P in new mineral forms that precipitate in their mycelium and assimilate P in organic forms that could be released after the death of the same fungus, as well.

The wide and varied P storage and release strategies of fungi could be exploited to overcome P limitation in agricultural soils, and reduce the need for phosphate fertilisers. In fact, the uptake of P in the mycelium both as organic forms and as secondary P-bearing biogenic minerals could provide to soil a slow-release source of P, that could be controlled by the requirements of plants roots and the community of soil decomposers.
